# Revisiting proboscidean phylogeny and evolution through total evidence and palaeogenetic analyses including *Notiomastodon* ancient DNA

**DOI:** 10.1016/j.isci.2021.103559

**Published:** 2021-12-04

**Authors:** Sina Baleka, Luciano Varela, P. Sebastián Tambusso, Johanna L.A. Paijmans, Dimila Mothé, Thomas W. Stafford, Richard A. Fariña, Michael Hofreiter

**Affiliations:** 1 Institute for Biochemistry and Biology, University of Potsdam, Karl-Liebknecht-Str. 24-25, 14476 Potsdam, Germany; 2 Faculty of Life and Environmental Sciences, University of Iceland, Sæmundargata 2, 102 Reykjavik, Iceland; 3 Departamento de Paleontología, Facultad de Ciencias, Universidad de la República, Iguá 4225, 11400 Montevideo, Uruguay; 4 Servicio Académico Universitario y Centro de Estudios Paleontológicos (SAUCE-P), Universidad de la República, Santa Isabel s/n, 91500 Sauce, Departamento de Canelones, Uruguay; 5 Laboratório de Mastozoologia, Instituto de Biociências, Universidade Federal do Estado do Rio de Janeiro, Av. Pasteur, 458/501, 22290-240 Rio de Janeiro, Brazil; 6 Programa de Pós-graduação em Biodiversidade e Biologia Evolutiva, Centro de Ciências da Saúde, Instituto de Biologia, Campus Ilha do Fundão, Universidade Federal do Rio de Janeiro, Rio de Janeiro, Brazil; 7 Stafford Research LLC, 200 Acadia Avenue, Lafayette, CO 80026-1845, USA

## Abstract

The extinct Gomphotheriidae is the only proboscidean family that colonized South America. The phylogenetic position of the endemic taxa has been through several revisions using morphological comparisons. Morphological studies are enhanced by paleogenetic analyses, a powerful tool to resolve phylogenetic relationships; however, ancient DNA (aDNA) preservation decreases in warmer regions. Despite the poor preservation conditions for aDNA in humid, sub-tropical climates, we recovered ∼3,000 bp of mtDNA of *Notiomastodon platensis* from the Arroyo del Vizcaíno site, Uruguay. Our calibrated phylogeny places *Notiomastodon* as a sister taxon to Elephantidae, with a divergence time of ∼13.5 Ma. Additionally, a total evidence analysis combining morphological and paleogenetic data shows that the three most diverse clades within Proboscidea diverged during the early Miocene, coinciding with the formation of a land passage between Africa and Eurasia. Our results are a further step toward aDNA analyses on Pleistocene samples from subtropical regions and provide a framework for proboscidean evolution.

## Highlights

• We assembled ∼3,000 bp of ancient DNA from a humid, sub-tropical environment
• Phylogenetic analysis places *Notiomastodon* as sister taxon to the Elephantidae
• Middle Miocene divergence between the lineage of *Notiomastodon* and the Elephantidae
• We present a phylogenetic framework for future studies on proboscidean evolution

## Graphical abstract

**Figure:**
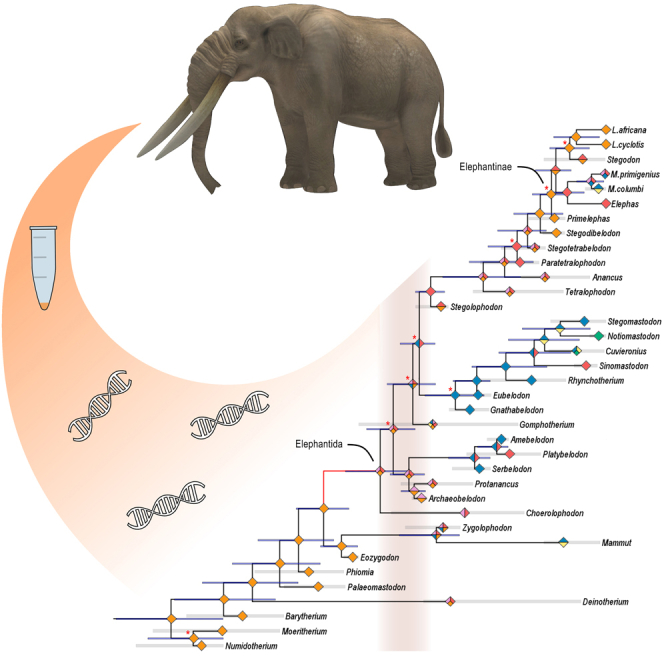

## Introduction

Proboscidea Illiger, 1811 is a mammalian order that includes the extant elephants (*Elephas* Linnaeus, 1758, and *Loxodonta* Anonymous, 1827) and a great diversity of extinct species (Shoshani and Tassy, 2005; Gheerbrant and Tassy, 2009). The order originated around 60 million years (Ma) ago in Africa (Gheerbrant, 2009). At present, the oldest proboscidean fossil is *Phosphatherium escuilliei* Gheerbrant, Sudre & Cappetta, 1996, from Morocco. It comprises cranial and mandibular elements dating to 55 Ma (Gheerbrant, 2009). The evolutionary history of proboscideans is marked by three major radiations. The first occurred during the late Palaeocene/Eocene, with the diversification of primitive proboscideans. The second radiation took place during the early Miocene, with the diversification of “Gomphotheriidae” Hay, 1922, (used *sensu lato* throughout the text, indicated by brackets) Mammutidae Hay, 1922, and Stegodontidae Osborn, 1918, a family within the Elephantoidea. The last radiation took place during the late Miocene/early Pliocene and resulted in the diversification of Elephantidae Gray (1821), as well part of the superfamily Elephantoidea, including the living elephants (Shoshani and Tassy, 1996; Gheerbrant and Tassy, 2009).

The “Gomphotheriidae” are considered the most diverse family within Proboscidea and include proboscideans with bunodont molars, i.e., molars with rounded cusps (Shoshani and Tassy, 2005). Despite their great diversity, the evolutionary relationships among the gomphotheres and the other Neogene lineages are still poorly understood, and “Gomphotheriidae” is usually recovered as paraphyletic, including Stegodontidae and Elephantidae (Shoshani and Tassy, 1996; Prado and Alberdi, 2008; Cozzuol et al., 2012). Gomphotheres occurred in Africa, Eurasia, and the New World and are the only proboscidean group that reached South America (Simpson and Paula-Couto, 1957; Ferretti, 2008; Mothé and Avilla, 2015). Studies on South American proboscideans started more than two centuries ago (Cuvier, 1806), and their taxonomic history is long and intricate. Recent studies of Ferretti (2008, 2010), Lucas (2008, 2013), and Mothé and colleagues (Mothé et al., 2012, 2017a, 2017b; Mothé and Avilla, 2015) reviewed the vast and abundant record of proboscideans from South America and updated their taxonomy, recognizing two monospecific taxa: *Cuvieronius hyodon* Osborn, 1933, and *Notiomastodon platensis* Ameghino, 1888.

Most of the phylogenetic studies of South American proboscideans are based on morphological data and recovered different possible combinations of evolutionary relationships among these brevirostrine (short jawed), bunodont proboscideans (Shoshani, 1996; Prado and Alberdi, 2008; Ferretti, 2010; Cozzuol et al., 2012; Mothé et al., 2013, 2016, 2017a). A molecular study (Buckley et al., 2019) based on proteomic analysis of bone collagen suggested that *Notiomastodon* would be more closely related to members of the Mammutidae (the family including *Mammut americanum*, the American true mastodon) than to the Elephantidae, as traditionally found in morphological studies. Also, Nogueira et al. (2021) recently recovered enamel peptides from a *N. platensis* molar from the Brazilian Intertropical Region, indicating that such peptides could be used as additional data for phylogenetic studies.

**Figure 1:**
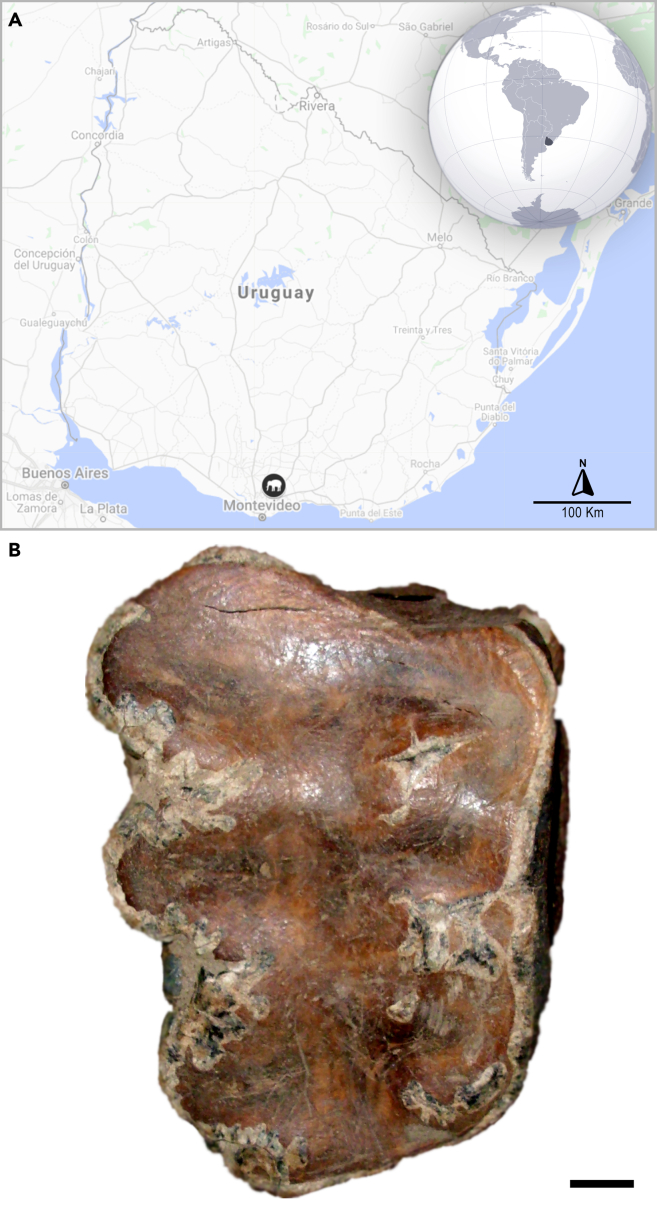
Overview over location and specimen (A) Location of the Arroyo del Vizcaíno site, where the specimen analyzed in this study was found. (B) First right upper molar of *Notiomastodon platensis* (CAV 499), in advanced wear stage, sampled for aDNA. Scale bar = 1 cm.

In this study, we present almost 3,000 base pairs of mitochondrial ancient DNA (aDNA) obtained from a specimen of *N. platensis* from Uruguay (Figure 1) and reconstruct a molecular phylogeny using these data together with previously retrieved DNA sequences from seven other members of the Proboscidea. Additionally, we combine molecular and morphological data to obtain a phylogenetic tree spanning all groups of the order Proboscidea.

## Results and discussion

### Ancient DNA authentication and capture efficiency

The biomolecular preservation of the sample was clearly near the limit for DNA sequencing. Also, the phylogenetic distance to previously analyzed species of Proboscidea made it challenging to retrieve sufficient sequence data for analysis. Despite extensive efforts, we were only able to recover slightly less than 3 kb of mitochondrial sequence. The poor preservation of the recovered DNA is exemplified by the characteristics of the reads identified as deriving from *Notiomastodon*. First, the read length is very short, with a mean of 34 bp and less than 65 reads longer than 50 bp (Figure S1B). Second, despite treatment with uracil glycosylase, the ends of the fragments show deamination rates of about 30% (Figure S1C). We do not see any evidence for the presence of nuclear mitochondrial DNA (NUMTs; e.g., heterozygosity) and could exclude the presence of frameshifts or stop codons in open reading frames.

**Figure 2:**
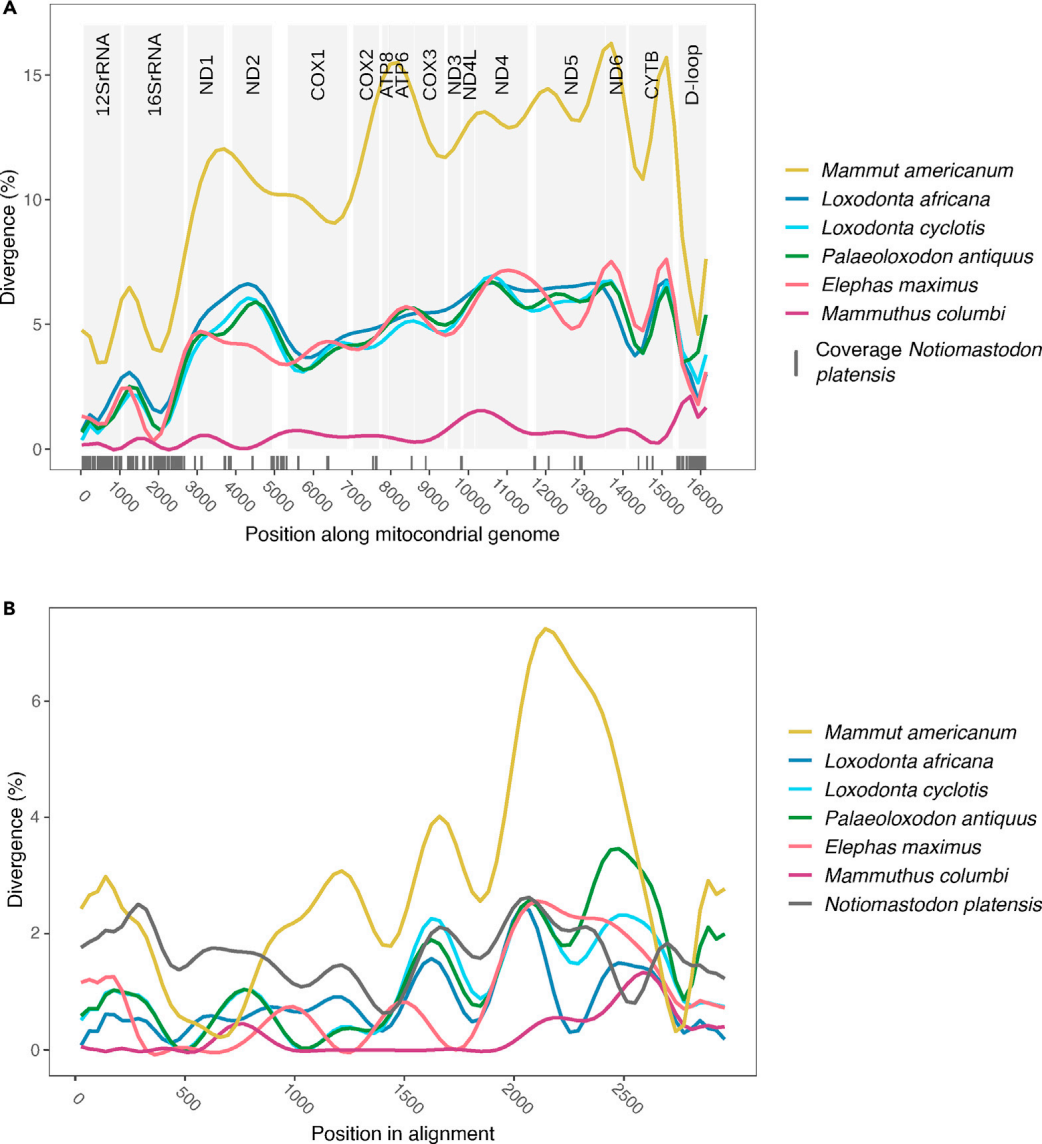
Pairwise sliding window comparison between different proboscidean species Shown is the percentage of divergence between *Mammuthus primigenius* (used as mapping reference; NC_007596) and the other color-indicated species. (A) Based on complete mitochondrial genomes, using a window size of 50-bp sliding in intervals of 5 bp, lines smoothed. Vertical gray lines show bases covered by our *Notiomastodon* consensus sequence. (B) Based on an alignment that includes only positions covered by our *Notiomastodon* consensus sequence, using non-overlapping windows of 5 bp, lines smoothed.

In addition to poor DNA preservation, previous work has shown that hybridization capture fails to recover sequences that are too divergent from the bait sequences (Paijmans et al., 2016, 2020). Since no sequences from close relatives are available, we used baits designed from members of the Elephantidae as well as the American mastodon. As is expected under such circumstances, we preferentially captured relatively conserved regions (Figure 2). Interestingly, for most of these regions, coverage was relatively high (Figure S1A). This is a direct result of the multiple libraries that were used to build the consensus sequence. Each library contains unique molecules and is therefore deduplicated separately, resulting in stacked reads in regions covered by more than one library. This suggests that it was more the genetic distance between baits and target, rather than a lack of mitochondrial target DNA in the extract, that prevented retrieval of a larger fraction of the mitochondrial genome. It has been shown that capturing mitochondrial genomes using a divergent bait sequence can lead to a loss of regions with more sequence divergence in favor of those with less sequence divergence (Paijmans et al. 2016, 2020; Portik et al., 2016, Peñalba et al., 2014). Additionally, variation in coverage has been observed in the capture of divergent species in previous studies (Paijmans et al., 2020). Divergence between bait and target does not seem to be the sole driver of this effect. Although the sequence divergence is expected to be significant between *Notiomastodon* and other proboscideans (divergence between the American mastodon and the woolly mammoth is between ∼3.5% and over 15%; Figure 2), successful capture has been reported for more divergent sequences (40%) in previous papers (e.g., Li et al., 2013). A previously proposed explanation for this large variation in coverage has been the bias introduced by amplification cycles or by the double-capture strategy (Paijmans et al., 2020).

Shotgun libraries contained from zero detectable mitochondrial reads to 0.0002% (Table S1). Despite the phylogenetic distance, the capture strategy was partially successful, leading to an at least 3.5× increase in mitochondrial content (endogenous mitochondrial DNA content for enriched samples used in constructing the consensus sequence was between 0.0009% and 0.0043%; both times mapped to *Loxodonta africana*; Table S1). It is unclear if changes in capture design would have resulted in higher enrichment, because highly divergent sequences have so far not been shown to profit from, e.g., lowering of hybridisation temperature (Paijmans et al., 2016). To the contrary, lowering the hybridisation temperature might prove counterproductive for libraries containing high levels of contamination (Paijmans et al., 2016). Additionally, due to the high levels of exogenous DNA in our libraries, both the relaxed and the iterative mapping approach we tried failed to yield interpretable results. As to our knowledge, no DNA sequences are so far available for the “Gomphotheriidae”, our analyses, nevertheless, represent another major step toward extending proboscidean paleogenetics from cold and temperate regions into warmer regions, where a number of recently extinct species and families are awaiting molecular analyses. There have been several recent publications reporting aDNA sequence data from tropical regions (e.g., Kehlmaier et al., 2017, 2021; Woods et al., 2018), but all these investigated samples are only a few thousand years old. While the geographic area in Uruguay itself is defined as humid sub-tropical, the sample age of ∼35 ka greatly extends the time range of previous studies. The fossils' depth-of-burial and the anoxic geochemical conditions most likely prevented total DNA loss that would be expected in warm climates.

Moreover, the high coverage of part of the mitochondrial genome suggests that sufficient ancient DNA is potentially available in samples from this site, at least for reconstructing mitochondrial genomes. As it may be difficult to design more efficient capture approaches, future studies might either target specific skeletal elements that are expected to have a better DNA preservation (e.g. Alberti et al., 2018, Pinhasi et al., 2015) or try to improve the ratio of endogenous to exogenous DNA during extraction and library prepration (e.g. Essel et al., 2021). As the approach using bleach prior to extraction (Korlević et al., 2015) was not successful in our case (see Table S1, libraries STE_B1_cap2 and STE_B2), targeting specific skeletal elements is more likely to yield improved DNA recovery.

### Phylogenetic reconstruction and divergence time estimates

**Figure 3:**
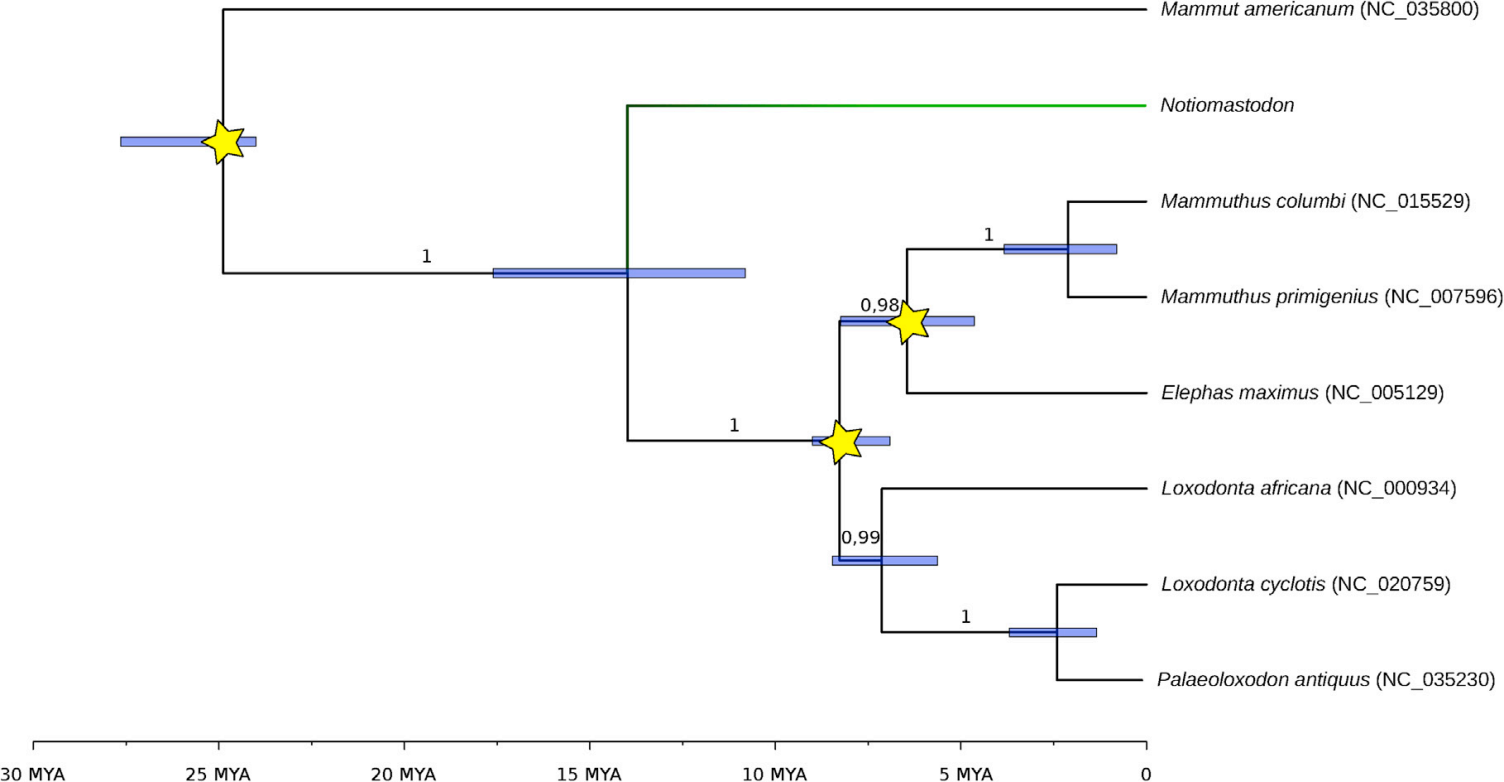
Fossil-calibrated Maximum Clade Credibility tree using published sequence data from seven proboscidean species as well as our reconstructed sequence of *Notiomastodon* Shown here is the tree reconstructed using all three node calibrations (yellow stars) with uniform prior distributions and “narrow” fossil calibrations from Brandt et al. (2012) (see Table S2 for other combinations of fossil calibrations and prior distributions). Blue bars represent 95% highest posterior probability intervals for node ages. Node support is given as Bayesian posterior probability

The 2,929-bp alignment used for calculating the calibrated tree still contained 152 polymorphic positions, 54 of which are parsimony informative. Our analyses place *Notiomastodon* as a sister taxon to the Elephantidae, a relation also found by Mothé et al. (2016) using morphological data for a “Gomphotheriidae” review. Despite the comparatively short length of the DNA sequence used for phylogenetic analyses (Rohland et al., 2007), support for a sister group relationship of *Notiomastodon* and Elephantidae is strong with a posterior probability of 1 (Figure 3) and a bootstrap value of 100 (Figure S1D). Moreover, the topologies recovered both for the seven Elephantidae species included in the Bayesian analysis and the 35 taxa in the maximum likelihood approach are identical to those obtained with full mitochondrial genomes (Figure S1D), except for the placement of Clade 1 mammoths within mammoth diversity, supporting the reliability of the topologies recovered with our dataset.

The phylogenetic position we recover—*Notiomastodon* being more closely related to Elephantidae—agrees with several morphological studies that recovered this South American proboscidean within the clade of trilophodont gomphotheres (Gheerbrant and Tassy, 2009; Shoshani, 1996; Shoshani et al., 1998, 2007) but disagrees with a recently published paleoproteomic study on the basis of collagen sequences (Buckley et al., 2019). In the latter study, *Notiomastodon* is placed closer to the Mammutidae, an unprecedented phylogenetic hypothesis. However, collagen is known to be a highly conserved biomolecule and provides only limited taxonomic resolution (Cappellini et al., 2018), an observation the authors emphasize as potentially limiting their analysis (Buckley et al., 2019).

Our molecular dating allows an estimation of the divergence time between *Notiomastodon* and the obtained sister lineage, the Elephantidae. We recover with about 13.5 Ma a much deeper divergence for *Notiomastodon* versus Elephantidae than for the most recent common ancestor of the Elephantidae, which is estimated to be around 8 Ma, again in agreement with previous results (Gheerbrant and Tassy, 2009; Mothé et al., 2016; Table S2). We tested the effect of different combinations of all three, two, or one internal fossil calibrations as well as the effect of the choice of calibration schemes and prior distribution. Exclusion of the oldest calibration point (split between the American mastodon and all other analyzed genera; Elephantid-Mastodon in Table S2) shifts the estimated divergence between *Notiomastodon* and the Elephantidae by ∼2–2.5 Ma to 11–12 Ma, which is to be expected, because this is the only calibration point that is older than the split between *Notiomastodon* and the Elephantidae. When all three calibration points were used, differences in calibration scheme (broad vs. narrow ages for fossil calibrations) as well as the choice of prior distribution (uniform, log-normal, normal) are not pronounced, with normal prior distributions being the most consistent between the two calibrations schemes (Table S2), whereas both log-normal and uniform prior calibrations differ by ∼300–400 ka between schemes. There was no apparent directional effect visible in the choice of prior distribution, e.g., using a log-normal prior distribution made the split younger when using all three as well as only the oldest calibration point, but older when using either the split between the African and the Eurasian lineages (*Loxodonta*-Eurasian in Table S2) or between the Asian elephants and the mammoths (Asian-mammoth in Table S2).

### Combining molecular and morphological data in a total evidence analysis

**Figure 4:**
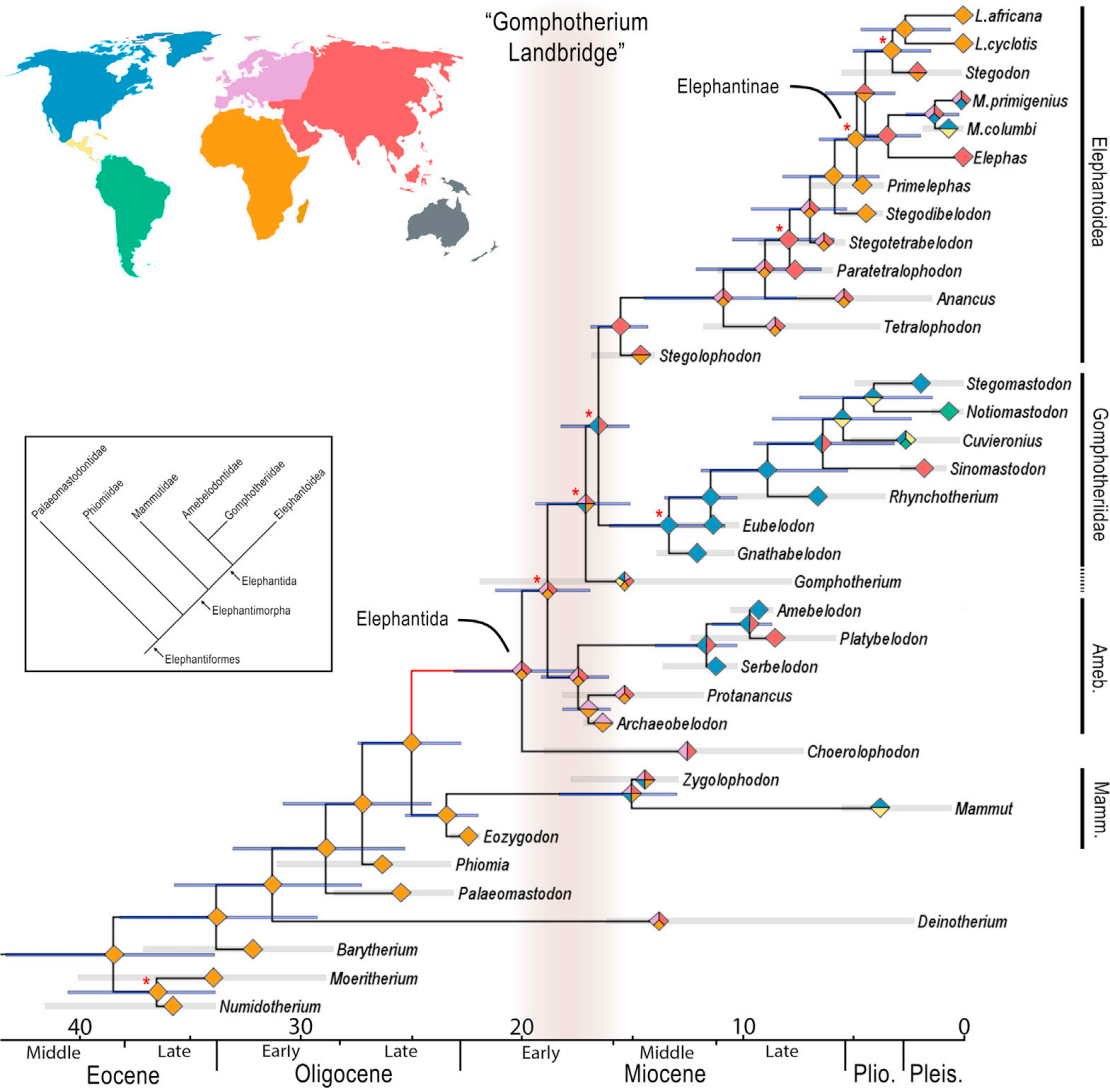
Total evidence phylogenetic inference and historical biogeography results See also Figure S2 for full node supports. Red asterisks show nodes with a posterior probability of less than 50. The red branch leading to Elephantida shows the zone of the tree where RPANDA detected a shift in the diversification dynamics. Blue bars represent age uncertainty. Gray bars represent taxa's stratigraphic ranges. Colors in the map correspond to the geographic areas defined in the biogeography analysis; diamonds at nodes show reconstructed ancestral areas based on this color scheme. Inset shows classical phylogeny of Proboscidea based on morphological characters. Ameb., Amebelodontidae; Mamm., Mammutidae

For the majority of fossil proboscideans, no aDNA sequence data are available because most species are beyond the age of the oldest aDNA presently recovered, an approximately 1.5-million-year-old mammoth specimen (van der Valk et al., 2021). Although there has been enormous progress analyzing ancient proteome data, reaching greater time depth than aDNA (Wadsworth and Buckley, 2014; Demarchi et al., 2016), data availability is limited and many extinct proboscidean species also exceed the time frame of proteome analyses, which is currently 2 million years for fossil enamel from temperate or tropical regions (Cappellini et al., 2019). Therefore, to obtain a better understanding of the relationships within the Proboscidea, we performed a total evidence analysis, combining the available DNA sequence data with a large morphological character matrix. The results of this analysis show a topology similar to previously published phylogenetic hypotheses of proboscidean evolution (Figures 4 and S2; Gheerbrant and Tassy, 2009; Shoshani, 1996; Shoshani et al., 2007, 1998). The recovered phylogeny shows an early diverging lineage related to the genus *Mammut* (family Mammutidae, the true mastodons) and the existence of three large groups concordant with the Amebelodontidae, “Gomphotheriidae”, and Elephantoidea. The results indicate relatively low support for the divergence times of these three commonly recognized groups, which do not allow to confidently resolve the relationships among them, with “Gomphotheriidae” still being paraphyletic, whereas the other two represent monophyletic groups. However, we performed additional analyses excluding two taxa that can be considered conflicting due to their probably paraphyletic nature—*Gomphotherium* and *Choerolophodon* (see Shoshani, 1996; Tassy, 1996; and Li et al., 2019 for discussions on the subject). The results obtained with this modified dataset showed an identical topology for the remaining taxa and much more support for the basal nodes, indicating that the topology obtained in the main test can be tentatively considered for further analyses (Figure S3). Regarding Elephantoidea, the analysis recovered *Stegodon* within the Elephantinae, although node support is low. This result is in contradiction with morphological parsimony results (Shoshani, 1996) that show *Stegodon* as forming a clade with *Stegolophodon* in the family Stegodontidae. In our results, *Stegolophodon* is placed as the most basal taxon in Elephantoidea, also contradicting parsimony results where it was placed within the clade. Finally, in relation to the clade including the trilophodont gomphotheres from Asia and the New World, the results are largely consistent with previous parsimony results (Shoshani, 1996), showing a clade comprising *Gnathabelodon*, *Eubelodon*, *Rhynchotherium*, *Sinomastodon*, *Cuvieronius*, *Stegomastodon*, and *Notiomastodon*. Our results show *Notiomastodon* as closely related to *Stegomastodon*, contrary to recent results by Mothé et al. (2016), but in agreement with the results of Shoshani (1996). However, it must be mentioned that the morphological data employed for the analysis are largely based on Shoshani (1996); new molecular and morphological data will be needed to better resolve the clade's phylogenetic relationships. Nevertheless, despite the long and complicated taxonomic history of these proboscideans, the North American species of *Stegomastodon* and the South American *Notiomastodon* are both valid and distinct taxa, according to recent morphological reviews (Mothé et al., 2012, 2017a, 2017b; Mothé and Avilla, 2015).

Additionally, we used this combined dataset for dating the proboscidean phylogeny. Regarding the obtained divergence times, the results were largely consistent with the molecular-only analysis. Interestingly, the results showed that the divergences of the three major groups, Amebelodontidae, “Gomphotheriidae”, and Elephantoidea, occurred during the early Miocene, ca. 18.9 Ma and 17.1 Ma (with a maximum span from 21.2 Ma to 15.1 Ma considering the 95% confidence range of both events). The later divergence (“Gomphotheriidae”-Elephantoidea) date is slightly older than the value obtained in the molecular-only analysis (∼13.5 Ma; Table S2), although their confidence intervals are largely overlapping. Also, previous research has shown that total evidence tip-dating and morphological clock approaches can provide slightly older dates, especially for basal nodes (O'Reilly et al., 2015; Puttick et al., 2016; Varela et al., 2019).

### Diversification and historical biogeography analysis

The diversification analysis conducted in RPANDA identified a single significant shift in the diversification dynamics located in the branch leading to the Elephantida (“Gomphotheriidae” and Elephantoidea) node and immediately before the divergence of the three major groups (Figure 3; BIC_random_/BIC_test_ = 4.36). Interestingly, this period is widely acknowledged as the time that a connection between Africa and Eurasia formed (∼16–19 Ma; Rögl, 1998; Harzhauser et al., 2007). This land passage, commonly known as “The *Gomphotherium* Landbridge,” allowed the passage of many mammals that were previously confined to Africa into Eurasia, including the Proboscidea (Sen, 2013; Rage and Gheerbrant, 2020). Regarding the New World brevirostrine gomphotheres, the node representing the last common ancestor of *Cuvieronius*, *Stegomastodon*, and *Notiomastodon* was dated to 5.49 Ma, similar to previous time estimates based on species' stratigraphic ranges (Mothé et al., 2016).

The historical biogeography analysis (Figures 4, S4, and S5) suggests that proboscideans may have left Africa only three times in phylogenetically distant clades (*Deinotherium* or Deinotheriidae, Mammutidae, and Elephantida). After the connection of Africa to Eurasia, a rapid global dispersal event is observed, which is related to the diversification shift obtained in the RPANDA analysis. These results match the fossil record for Proboscidea, where the first undisputed members (*Prodeinotherium* and *Gomphotherium*) of the clade outside Africa are recorded in Pakistan during the Late Oligocene-Early Miocene (Sen, 2013). Dispersal from Eurasia back into Africa happened even less often, potentially in the lineage ancestral to Elephantidae. There is also some evidence for additional dispersal between Africa and Eurasia leading to the hybrid origin of the European straight-tusked elephant (Palkopoulou et al., 2018), but when and in which direction this occurred is unknown. At least two large vicariance events are observed, one among the ancestors of the extant genera *Elephas* and *Loxodonta* and another marking the arrival of the ancestor of *Notiomastodon* to North America. Proboscideans apparently reached the Americas independently at least three times, with the ancestors of the American mastodon (Mammutidae), the shovel-tusked Amebelodontidae, and the mammoths (Elephantidae). There is also evidence for repeated Pleistocene mammoth dispersal between Eurasia and North America (Debruyne et al., 2008; van der Valk et al., 2021).

Finally, the dispersal to South America was modeled differently when considering the two age constraints for the formation of the Isthmus of Panama. When the age constraint was set to 3.5 Ma, two dispersal events were recorded, one for *Cuvieronius* and the other for *Notiomastodon* (Figure S4). When the constraint was set to 9 Ma, a single dispersal to South America as early as ∼5.5 Ma was obtained for the common ancestor of *Cuvieronius*, *Notiomastodon*, and *Stegomastodon* (Figure S5). The earliest undisputed record of a proboscidean in South America is at 2.5 Ma in Argentina (Reguero et al., 2007). This age is consistent with using the late age constraint. Nonetheless, the controversial *Amahuacatherium* from the Late Miocene of Peru could represent an early dispersal event coinciding with an early “Great American Biotic Interchange” (GABI) that is compatible with the 9-Ma constraint (Campbell et al., 2000, 2009). However, several arguments regarding the validity of its diagnostic features were recently published (Alberdi et al., 2004; Ferretti, 2008; Mothé and Avilla, 2015). Consequently, the validity of *Amahuacatherium* is still questionable, with some authors considering the specimen as possibly belonging to *Notiomastodon* (Mothé and Avilla, 2015) and probably originating from the Pleistocene (Alberdi et al., 2004; Ferretti, 2008; Lucas, 2013).

Considering this, a late arrival scenario is more plausible for South American gomphotheres. The GABI was probably a complex, multi-phase process, with many intervening factors of differential importance in each clade (O'Dea et al., 2016; Jaramillo, 2018). In the case of proboscideans, southward emigration must have occurred at a late stage. As long-distance swimming is known for modern elephants (Johnson, 1980) and presumed for other taxa within the clade, gomphothere's late arrival in South America may be due more to unsuitable environmental conditions before the Pliocene and Pleistocene (Jaramillo, 2018) than to lack of a continuous land bridge.

## Conclusions

Our analysis of aDNA sequences from a 35,000-year-old fossil from a Uruguayan site marks a further step forward in the retrieval of Pleistocene aDNA from humid subtropical regions. Given the numerous and often unique, extinct species from warm climatic regions, our results portend new possibilities for a better understanding of these species' evolutionary history. Our results also show that because living and late Pleistocene representatives (i.e., those that are within the temporal window of DNA preservation) are a relatively small proportion of the taxa of any animal group, total evidence analysis is a useful and underappreciated tool for revealing evolutionary processes and events on a larger temporal scale. Our application of this approach to the order Proboscidea allows insights into their evolution dating back almost 40 Ma and resolves most of their divergence events. However, some internal phylogenetic relationships still remain unresolved in our analyses, especially regarding several representatives of the “Gomphotheriidae”. Nevertheless, our resolution of the evolutionary and biogeographical history of *N. platensis*, the unique, endemic proboscidean species of South America, substantially advances our understanding of the natural history of this group in the New World.

### Limitations of the study

Our study is limited by representing a single individual of *N. platensis* and by the fact that we were only able to recover partial mitochondrial data. Additionally, phylogenies recovered from mtDNA can differ from the species tree. The divergence times we recover using mitochondrial DNA are estimates, and new fossil finds could alter our calibration scheme and therefore change our current divergence time estimates. Additionally, a limitation of our study in regard to the total evidence analysis is the low diversity of bunodont proboscideans with recovered aDNA—only *Notiomastodon—*considering their high diversity and the wide geographic distribution of the group. Considering the approach to the historical biogeography analysis we have taken, it has to be noted that the recognition of small-scale events (species level) is limited. Additionally, as with the divergence time estimates, new fossil finds could change the picture currently seen in the historical biogeography analysis.

## STAR★Methods

### Key resources table

**Table:** 

REAGENT or RESOURCE	SOURCE	IDENTIFIER
**Chemicals, peptides, and recombinant proteins**		

Guanidine hydrochloride	Roth	Cat#0037.1
QIAGEN MinElute kit	Qiagen	Cat#28004

**Critical commercial assays**		

D1000 screen tape (Tapestation2200)	Agilent	Cat#5067-5582
dsDNA HS assay kit (qubit 2.0)	Thermofisher	Cat#Q32851

**Deposited data**		

Incomplete mtDNA sequence of *Notiomastodon platensis* (GenBank accession number)	This paper	GenBank: OL631592-OL631597
Unprocessed sequence data, fastq format (NCBI SRA bioproject PRJNA771914)	This paper	SRA: SRR16498912-SRR16498930
Additional supplemental material	This paper	https://doi.org/10.17632/msbxvxsdnh.1

**Oligonucleotides**		

CL9 extension primer: GTGACTGGAGTTCAGACG TGTGCTCTTCCGATCT	Gansauge and Meyer, 2013	Sigma Aldrich
Double-stranded adapter Strand 1 (CL53): CGACGCTCTTC-ddC (ddC = dideoxycytidine) Strand 2 (CL73): [Phosphate]GGAAGAGCGT CGTGTAGGGAAAGAG∗T∗G∗T∗A (∗ = phosphothioate linkage)	Gansauge and Meyer, 2013	Sigma Aldrich
CL78: AGATCGGAAG[C3Spacer] _10_ [TEG-biotin] (TEG = triethylene glycol spacer)	Gansauge and Meyer, 2013	Sigma Aldrich
P5 indexing primer: AATGATACGGCGACCACCG AGATCTACACnnnnnnnnACACTCT TTCCCTACACGACGCTCTT	Gansauge and Meyer, 2013	Sigma Aldrich
P7 indexing primer: CAAGCAGAAGACGGCATAC GAGATnnnnnnnnGTGACTGGAGTTCAGACGTGT	Gansauge and Meyer, 2013	Sigma Aldrich

**Software and algorithms**		

Cutadapt v1.10	Martin, 2011	https://cutadapt.readthedocs.io/en/stable/
FLASH v1.2.11	Magoč and Salzberg, 2011	https://ccb.jhu.edu/software/FLASH/
BWA v0.7.8	Li and Durbin, 2009	http://bio-bwa.sourceforge.net/
Samtools v1.1.19	Li et al., 2009	https://sourceforge.net/projects/samtools/files/samtools/
MarkReadsByStartEnd.jar	NA	https://github.com/dariober/Java-cafe/tree/master/MarkDupsByStartEnd
MapDamage v2.0.2	Jónsson et al., 2013	https://ginolhac.github.io/mapDamage/
Geneious v10.1.3	Kearse et al., 2012	https://www.geneious.com/
MITOS	Bernt et al., 2013	http://mitos.bioinf.uni-leipzig.de/index.py
MITObim v1.9.1	Hahn et al., 2013	https://github.com/chrishah/MITObim
PartitionFinder v1.1.1	Lanfear et al., 2012	https://www.robertlanfear.com/partitionfinder/
ggplot2	Wickham, 2011	https://ggplot2.tidyverse.org/
RStudio	RStudio Team, 2019	https://www.rstudio.com/
MAFFT v7.453	Katoh and Standley, 2013	https://mafft.cbrc.jp/alignment/software/
RAxML-HPC 8.2.4	Stamatakis, 2014	https://cme.h-its.org/exelixis/web/software/raxml/
BEAST v1.8.4	Drummond et al., 2012	https://beast.community/
Tracer v1.6	Rambaut et al., 2014	http://beast.bio.ed.ac.uk/Tracer
Treeannotator	NA	https://beast.community/treeannotator
FigTree v1.4.2	NA	http://tree.bio.ed.ac.uk/software/figtree/
BEAST2 v2.3.2	Bouckaert et al., 2014	https://www.beast2.org/
BEASTMasteR	Matzke, 2015	https://github.com/nmatzke/BEASTmasteR
RPANDA	Morlon et al., 2016	https://github.com/hmorlon/PANDA
BioGeoBEARS	Matzke, 2013	https://github.com/nmatzke/BioGeoBEARS

**Other**		

Proteinase K	Promega	Cat#V3021
Zymo-spin V column extension reservoir	Zymo	Cat#C1016-50
Circligase II	Biozym	Cat#131402(CL9021K)
Uracil-DNA glycosylase (afu UDG)	NEB	Cat#M0279S
FastAP	Thermo Fisher	Cat#EF0651
Dynabeads MyOne C1	Thermo Fisher	Cat#65001
Bst 2.0 polymerase	NEB	Cat#M0537S
T4 DNA Polymerase	Thermo Fisher	Cat#EP0061
Buffer tango (10×)	Thermo Fisher	Cat#BY5
T4 DNA ligase	Thermo Fisher	Cat#EL0011
Accuprime Pfx	Thermo Fisher	Cat#12344024
PEG-4000	Thermo Fisher	Cat#EP0061
SYBR green PCR MasterMix	Thermo Fisher	Cat#4309155
HI-RPM hybridization buffer (2×)	Agilent	Cat#5190-0403
Blocking agent (1×)	Agilent	Cat#5188-5281

### Resource availability

#### Lead contact

Further information and requests should be directed to and will be fulfilled by the lead contact, Sina Baleka (sina.baleka@gmail.com).

#### Materials availability

This study did not generate new unique reagents.

### Experimental model and subject details

#### Geography and site Geology

The tooth analysed for mitochondrial aDNA sequences is from the Arroyo del Vizcaíno site (Fariña et al., 2014), a palaeontological locality near the town of Sauce in southern Uruguay (34º37′3 "S, 56º2′33″ W; Figure 1A). The site was discovered and excavated in 1997 and again in 2011 and 2012 (Fariña, et al., 2014), as well as on later occasions. The fossils occur in alluviated microbasins or plunge pools cut into more easily eroded zones of the bedrock, Cretaceous Mercedes Fm. silicified sandstone. Approximately 1 m of alluvium fills the 11-meter long (W-E) basin. The basal stratum, Bed 1, lies unconformably on the Cretaceous sandstone and is green muddy sand that is not fossiliferous. Lying unconformably above is the highly fossiliferous Bed 2, which consists of basal, muddy sandy gravels that fine-upward into muddy sands. The uppermost stratum, Bed 3, forms the modern stream bed and comprises modern, reworked Bed 2 sediments and occasional Bed 2 bones.

The bones in Bed 2 are single, in most cases disarticulated cranial and post-cranial skeletal elements, and rarely isolated teeth, belonging to the impressive South American megafauna (Fariña et al., 2013). Excavations have yielded >2000 skeletal elements, with hundreds more remaining *in situ.* There are over 40 individual animals representing 16 different taxa, with the majority (∼94%) of fossils being from the giant ground sloth*, Lestodon armatus.* Other megafauna recovered include *Glossotherium robustum*, *Mylodon darwinii*, *Doedicurus clavicaudatus*, *Panochthus tuberculatus*, *Glyptodon reticulatus*, *Toxodon platensis*, *Smilodon populator*, *Hippidion principale*, indeterminate Cervidae and Camelidae, and the proboscidean *Notiomastodon platensis* that was studied in this paper.

Fossils in Bed 2 were previously ^14^C dated to 27.0–30.1 ka RC by three labs using wood and bone (Fariña, et al., 2014). Six ^14^C dates on *Lestodon armatus* bone collagen ranged from 27,000 ± 450 (URU-0496) to 30,100 ± 600 (URU-0493). A *Panochtus* sp. scute dated 29,220 ± 300 RC yr. (URU-0574). Stratigraphically associated wood dated 29,150 ± 320 (URU-0562) and 29,350 ± 315 RC yr (URU-0561; Fariña, et al., 2014). Based on these initial data, the calibrated age range is 30.2–35.9 ka cal BP.

The age estimate for Bed 2 fossils was reassessed using improved collagen purification techniques, i.e., ultrafiltration, which isolates >30,000 molecular weight (>30 kDa) gelatinized collagen (Figure S6). These new ^14^C dates on bone from *Lestodon, Smilodon,* and *Notiomastodon* provide a more accurate date range of 29.9 to 30.7 ka RC years for the bone bed and a direct age of 30,510 ± 240 RC yr (UCIAMS-142845) for the *Notiomastodon platensis* tooth used for aDNA analyses (Figure S6). Until a larger number of taxa are dated, the calibrated age range of Arroyo del Vizcaíno fossils is assumed to be 34.8 to 35.6 ka cal BP.

#### Specimen description

The *Notiomastodon* tooth analysed in this report was a single, isolated 1^st^ right upper molar (RM^1^) from the middle of Bed 2, completely surrounded by a fine matrix and in proximity to a sloth tibia. The tooth is one of three *Notiomastodon* skeletal elements found in Bed 2.

The tooth was very use-worn (Figure 1B; wear stage 4 of Simpson and Paula-Couto, 1957; Mothé et al., 2010). It presents the typical dental features of South American proboscideans, such as the bunodont morphology (blunt and rounded pairs of cusps, the lophs), three lophs (since it is a molar from intermediate dentition), and a distal cingulum composed of several small accessory cusps (Figure 1B). The roots were not preserved. The crown structures, e.g., the main and secondary cusps and the accessory cusps have largely eroded during mastication (Shoshani and Tassy, 1996; Mothé and Avilla, 2015). Enamel traces are still present on the labial and lingual borders, and on the labial interloph regions. According to the type of molar (RM^1^) and its wear stage, we inferred that it belonged to an adult individual of *Notiomastodon platensis*, possibly around 20 years of age (Mothé et al., 2010). The specimen is archived at the SAUCE-P Laboratory and Collection under the collection number CAV 499, Sauce, Uruguay.

### Method details

#### DNA extraction and library preparation

All palaeogenetic work was carried out in designated ancient DNA facilities at the University of Potsdam. Pieces of the dentine were ground to powder using mortar and pestle or a microdismembrator at a frequency of 30 Hertz for 10 sec. Nineteen extracts were prepared, each using approximately 50 mg total of dentine powder, and extracted following the protocol of Dabney et al. (2013). Six of these extracts were obtained after the powder had been treated with 1 mL of 1% sodium hypochlorite for 15 mins prior to extraction (Korlević et al., 2015). All samples were incubated overnight in 1 mL extraction buffer (0.45 M EDTA, 0.25 mg/mL Proteinase K) at 37°C under constant rotation. Next, undigested material was pelleted through centrifugation and the supernatant was transferred into 13 mL of binding buffer (5 M guanidine hydrochloride, 40% isopropanol, 0.05% Tween-20, and 90 mM sodium acetate), which was then passed through QIAGEN MinElute columns fitted with a reservoir (Zymo-Spin V). The mix of supernatant and binding buffer for samples STE_CO3 and STE_CO4, as well as STE_UV5 and STE_UV6 were run through the same purification column, respectively, in an attempt to get higher concentrations in the final, purified extracts (Table S1). This was followed by two subsequent wash steps with PE buffer (QIAGEN) and a dry spin of 1 min at 13,000 rpm. The purified DNA was eluted in 25 μL TET buffer (10 mM Tris-HCl, 1 mM EDTA, 0.05% Tween-20).

All seventeen extracts were converted into single-stranded libraries including UDG treatment to reduce DNA damage (Gansauge and Meyer, 2013). To remove residual phosphate groups, one Unit of FastAP was used. Then the DNA was denatured at 95°C for 2 min, resulting in single-stranded DNA. Adaptor CL78 was now ligated to the 3′ end in an 80 μL reaction containing 20% (vol/vol) PEG-4000, 0.125 mM CL78, and 2.5 U/μL Circligase II, which was incubated overnight at 60°C. The DNA was immobilised on streptavidin covered magnetic beads (Dynabeads MyOne C1) and extension primer CL9 was annealed to the complementary CL78 adaptor. To fill in the second strand, a 50 μL reaction with the following reagents was used: 1× isothermal amplification buffer, 250 mM of each dNTP, 2 mM CL9 extension primer, and 0.48 U/μL Bst 2.0 polymerase. This was followed by a 100 μL reaction to achieve blunt-ended molecules that contained 1× Buffer Tango, 0.025% (vol/vol) Tween 20, 100 mM of each dNTP, and 0.05 U/μL T4 DNA polymerase. In a 100 μL reaction containing 1× T4 DNA ligase buffer, 5% (vol/vol) PEG-4000, 0.025% (vol/vol) Tween 20, 2 mM double-stranded adaptor, and 0.1 U/μL T4 DNA ligase, the second, double-stranded adaptor (CL53/CL73) was ligated to the molecules. As before, we incubated the mixture at 95°C for 1 min to denature the DNA molecule and the strand complementary to the original single-stranded molecule was eluted in 25 μL of TET buffer. Libraries were amplified and double-indexed in 80 μL reactions containing 1× AccuPrime Pfx reaction mix, 10 mM each of P5 and P7 indexing primers, and 0.025 U/μL AccuPrime Pfx polymerase. The optimal number of cycles was determined by qPCR prior to amplification. This was done in 10 μL reactions containing 1× SYBR green qPCR master mix, 0.2 mM each of IS7 and IS8 amplification primers, and 1 μL of a 1:20 dilution of the unamplified library.

#### Enrichment and sequencing

Twelve libraries were arbitrarily chosen for enrichment of mitochondrial DNA (mtDNA) using the same capture probes as described in Meyer et al. (2017), but following the protocol described in Gonzalez-Fortes and Paijmans (2019). Library and bait were pooled in a 10:1 ratio, mixed with 2 μM of each blocking oligonucleotide, 1× HI-RPM hybridization buffer and 1× Blocking Agent. We performed two subsequent rounds of enrichment, each time starting at 95°C for 5 min and then cooling down to 65°C at 0.1°C/sec, holding the temperature at 65°C for ∼24 hours. The capture reactions included extraction and library blanks as well as additional capture blanks.

The 12 enriched libraries and 5 shotgun libraries were sequenced on an Illumina NextSeq 500 as described in Paijmans et al. (2017), either in single end mode (SE, 1 x 75 cycles) or in paired end mode (PE, 2 x 75 cycles). To check for the presence of contamination, extraction and library blanks were included both in shotgun sequencing as well as after enrichment.

### Quantification and statistical analyses

#### Data processing and consensus calling

Adapter sequences and low-quality bases (-q 30) were trimmed using the program cutadapt 1.10 (Martin, 2011). Sequences shorter than 25 bp were removed (-m 25). FLASH 1.2.11 (Magoč and Salzberg, 2011) was used to merge paired end sequencing data with a minimum overlap of 15 bp (-m 15) and a maximum of 10% difference allowed (-x 0.1).

Reads were mapped against the genome of the African Savannah elephant *Loxodonta africana* (loxAfr3, GenBank Assembly ID: GCA_000001905.1), the mitochondrial genome of the same species (GenBank: NC_000943) as well as mitogenomes of *Mammuthus primigenius* (GenBank: NC_007596) and *Mammut americanum* (GenBank: NC_009574) using BWA 0.7.8 with default parameter values (Li and Durbin, 2009). Samtools 0.1.19 (Li et al., 2009) was used to remove reads with a mapping quality less than 30 (-q 30). Duplicate reads originating from PCR amplification were identified using MarkDuplicatesByStartEnd.jar (https://github.com/dariober/Java-cafe/tree/master/MarkDupsByStartEnd) and removed using samtools. Mapping to the different mitochondrial references showed only few differences in the number of reads mapping. However, most reads were recovered when mapping to the mitochondrial genome of *M. primigenius*. Therefore, after deduplication, reads mapped to *M. primigenius* from all 10 enriched libraries (excluding STE_B1 and STED2, which showed extremely low numbers of reads mapping to the mitochondrial genome after deduplication) were merged with samtools and analysed using the program MapDamage 2.0.2 (Jónsson et al., 2013) to confirm the presence of age-specific damage patterns.

A consensus sequence was called using Geneious 10.1.3 (http://www.geneious.com, Kearse et al., 2012) with an 85% majority rule for base calling and a minimum coverage of 3. The high consensus threshold excludes the possibility of SNPs deriving from damage patterns or nuclear mitochondrial DNA (NUMTs), which would show signs of heterozygosity in contrast to the haploid mitochondrial DNA. Additionally, we complemented the missing parts in our *Notiomastodon* consensus sequence with the reference sequence of the woolly mammoth and used this “hybrid” sequence to test for frameshifts or in-frame stop codons using the webserver MITOS (Bernt et al., 2013).

Since there is no reference from within the same family (‘Gomphotheriidae’) available, both a relaxed mapping (bwa -n 0.01) and an iterative mapping approach using MITObim 1.9.1 (Hahn et al., 2013) as described in Westbury et al. (2017) were tried in the attempt to recover regions that are more distant from Elephantidae.

#### Competitive mapping to human mtDNA

To exclude the presence of human derived reads, we remapped all reads previously mapped to the woolly mammoth reference sequence (GenBank: NC_007596) to a concatenated sequence of the former and the human mitochondrial reference sequence (GenBank: NC_012920) in a competitive mapping approach (Feuerborn et al., 2020), which allows for the identification of human DNA in our mapped reads. 74 reads (3.35%) mapped better to the human reference sequence, but we could exclude any human-derived substitutions in our *Notiomastodon* consensus sequence due to our strict consensus calling approach.

#### Pairwise distance analysis

We calculated pairwise divergences between the *Mammuthus primigenius* reference sequence (NC_007,596, belonging to mitochondrial clade 1, haplogroup D&E (Debruyne et al., 2008); since this was used as mapping reference for our *Notiomastodon* consensus sequence) and six proboscidean mitogenomic reference sequences (*L. africana* and *M. americanum* as used during mapping along with *Loxodonta cyclotis* (GenBank: NC_020759), *Palaeoloxodon antiquus* (GenBank: NC_035230), *Elephas maximus* (GenBank: NC_005129), and *Mammuthus columbi* (GenBank: NC_015529), belonging to mitochondrial clade 1, haplogroup F (Enk et al., 2011)) to get an overview of sequence divergence across the mitochondrial genome (Figure 2). Sequences were aligned using the --auto option in MAFFT 7.453 (Katoh et al., 2002; Katoh and Standley, 2013) and we calculated pairwise divergence in sliding windows of 50 bp length at 5 bp intervals using a custom Perl script. Plotting was done using ggplot2 (Wickham, 2011) in RStudio (RStudio Team, 2019) and lines were smoothed (Figure 2A).

We used the same alignment as above, added our *Notiomastodon* consensus sequence and removed all columns with missing data resulting in an alignment of 2940 bp. We then calculated pairwise divergence in non-overlapping 5bp windows and again plotted the data as described above (Figure 2B).

#### Molecular phylogenetic analysis

The consensus sequence for *Notiomastodon platensis* was aligned to seven proboscidean mitochondrial reference sequences as described above and columns with missing data were removed from the alignment, resulting in a final length of 2929 bp.

A Bayesian analysis was performed in BEAST 1.8.4 (Drummond et al., 2012), in order to estimate the divergence time between *Notiomastodon platensis* and the Elephantidae. An appropriate partitioning scheme from all possible combinations of available genes, two rRNAs, tRNAs, and part of the D-loop was selected using PartitionFinder 1.1.1 under the Bayesian Information Criterion (Lanfear et al., 2012). Because of the incomplete coverage of our sequence, not all genes and tRNAs were available and we did not distinguish individual coding positions for genes. This resulted in only one partition with the TrN+I substitution model. We applied a speciation birth-death tree prior and tested for rate variation by using a lognormal relaxed clock. Since the variation of the substitution rate among lineages was found to abut zero, we reran the analysis with a strict clock and applied this to all successive runs.

To calibrate the molecular clock, we ran several analyses using either narrow fossil calibrations (Brandt et al., 2012) or broad fossil calibrations (Rohland et al., 2010). Detailed justifications for the broad fossil calibration scheme can be found in Supplementary Text S5 in Rohland et al. (2010) and are mainly based on Sanders et al. (2010). Narrow fossil calibrations are based on the broad fossil calibration scheme but exclude questionable taxa and assume monophyly for *Elephas* and *Loxodonta*, which is not given within the broad fossil calibration scheme (Rohland et al., 2010; Brandt et al., 2012). We calibrated up to three internal nodes, using all combinations of one, two or all three calibration points. Prior distributions for fossil priors were drawn from uniform, lognormal or normal distributions (see Table S2). The Markov Chain Monte Carlo (MCMC) was run for 10 million generations each, sampling every 1000 generations. Chain convergence and appropriate posterior sampling of all parameters (ESS >200) were examined in Tracer 1.6 (Rambaut et al., 2014; available from http://beast.bio.ed.ac.uk/Tracer). The first 10% of trees were removed as burn-in from each run, trees were summarized and maximum clade credibility (MCC) trees identified using the program TreeAnnotator 1.8.4, which is distributed as part of the BEAST package. MCC trees were visualized using FigTree 1.4.2 (available from http://tree.bio.ed.ac.uk/software/figtree/).

Additionally, we used a maximum-likelihood approach on a bigger data set to reconstruct the phylogenetic position of *Notiomastodon*. Thirty-five proboscidean mitochondrial sequences (see Figure S1D for GenBank accession numbers) were aligned as described before. From this alignment (excluding *Notiomastodon*) we calculated a maximum-likelihood tree using RAxML-HPC 8.2.4 (Stamatakis, 2014) CIPRES black box version on the CIPRES Science Gateway (Miller et al., 2010), with the default GTR+CAT substitution model in place. This phylogeny of complete mitochondrial genomes (16,026 bp) was used in comparison to a phylogeny that included the incomplete sequence of *Notiomastodon* and therefore reduced the length of the alignment. We added the sequence of *Notiomastodon platensis* to the alignment using MAFFT as described before and removed missing data. This resulted in an alignment of 2548 bp length, which is shorter than the original consensus sequence of *Notiomastodon*. This reduction in the length of the alignment is caused by the presence of missing data in many published woolly mammoth mitochondrial sequences. A maximum-likelihood tree was calculated as described before and the recovered topologies were compared for consistency.

##### Total evidence phylogenetic analysis

We performed a total evidence approach by combining the new molecular data with published morphological data for a wide range of proboscidean fossil taxa. We updated the morphological matrix of Shoshani (1996) and Shoshani et al. (2006), and added recent data for several of the included taxa (See Supplemental information). The morphological data matrix covers 36 taxa from all continents and spans the entire Cenozoic. The matrix contains 125 characters, 101 cranial and 24 postcranial, of which 96 are multistate; none was treated as ordered. The basal proboscidean *Moeritherium* was used as the outgroup.

We used Bayesian methods that allow for the simultaneous estimation of topology and divergence times, including fossil taxa as tips in a tip-dating approach. Several studies have shown that Bayesian methods combining molecular and morphological data and including fossil taxa, even when missing data is present in several taxa, are useful for obtaining accurate phylogenies (Wiens, 2003, 2009; Wiens et al., 2010; Guillerme and Cooper, 2016; Mongiardino Koch et al., 2021). Specifically, we used a Birth-Death with Serial Skyline Sampling (BDSKY) tree prior (Stadler et al., 2013) with constant sampling over time, thus analogous to a BDSS tree prior (Stadler, 2010) or a fossilized birth-death (FBD) model (Heath et al., 2014). This analysis was performed with the software BEAST2 v2.3.2 (Bouckaert et al., 2014) using BEASTMasteR (Matzke, 2015) for the assembly of the xml file. For the morphological data, Lewis's (2001) Mkv model was used for the analysis. Variable gamma rates were favoured over equal rates (log BF > 200; stepping-stone sampling) after the implementation of the stepping stone method. Regarding the molecular data, due to limitations of the available models, the GTR nucleotide substitution model was used. A lognormal uncorrelated relaxed clock model was used for the analysis and uninformative uniform distribution [0,10] hyperpriors were used for the mean and the standard deviation. Additionally, birth and death rate priors were set to uniform distributions [0,100] and the sampling rate prior was also set to a uniform distribution [0,10]. Considering the extant taxa sampled in our dataset, Rho was fixed to one.

Biostratigraphic ranges of fossil taxa are provided in Table S3. All fossil taxa were treated as tips and calibrated with uniform distributions to account for stratigraphic uncertainty (Wood et al., 2013). Also, specific basal nodes were calibrated similar to the previous molecular-only analysis. The analysis was run for 100 million generations with sampling every 1000 generations. Convergence and effective sample size (ESS) were checked with Tracer v1.6. Finally, MCMC results were summarized in an MCC tree discarding the first 25% of trees as burn-in.

##### Diversification analysis

We performed a diversification analysis over the total evidence phylogeny to investigate any possible shifts in diversification during the history of the clade. For this purpose, we used the R package RPANDA (Morlon et al., 2016). RPANDA allows for the detection of diversification shifts in regions of a tree that have distinct branching patterns, using a model-free approach to efficiently summarize the shape of a phylogenetic tree by its spectral density. In particular, we used the function spectR to detect the optimal configuration of shifts based on the eigenvalues associated with the phylogeny and characteristics associated with the spectrum of eigenvalues. Once the optimal shift configuration was obtained, we used the BICompare function to map the shifts on the phylogeny, and chose the best configuration for the previously obtained number of shifts comparing the BIC values. Considering that our total evidence phylogeny was non-ultrametric due to the inclusion of fossil taxa, we used the argument “non-ultrametric” for the analysis. This approach allowed us to obtain the optimal number of shifts recognized for the data and also select the best position for those shifts based on a statistical approach.

##### Historical biogeography

We also performed a biogeography analysis using the currently available data to trace the evolutionary history of proboscideans geographically and chronologically. Reconstruction of ancestral distributions was based on the time-calibrated phylogeny. Because biogeographic models assume that processes happen at the species level, and using genera ranges at tips could result in invalid results, our analysis used the ranges of the most widely distributed species in each genus (Table S3). This approach represents clear limitations for the results of the analyses, mainly regarding the recognition of small-scale events and, especially, those occurring at the species level. In this regard, it should be made clear that our goal is focused on reconstructing the deeper and most important biogeographic events, therefore ignoring the potential events that could have occurred in relation to specific extinct species within genera (other than the most widely distributed one for the respective genus) or other yet-unknown extinct species. Therefore, our analyses focused on evaluating the most probable areas of origin of the different proboscidean clades, with special emphasis on their biogeographic distribution in the Americas. We defined six geographic areas: Africa, Europe, Asia, North America, Central America, and South America. The biogeographic distribution of proboscideans was modelled in the R package BioGeoBEARS (Matzke, 2013) using the DEC model, since this model permitted speciation via the biologically relevant mechanisms of widespread vicariance and subsequent sympatry (Givnish et al., 2016). Each taxon was assigned to a set of these areas. The maximum number of areas at a given node was set to five, which is the range of the taxon present in the largest number of areas (*Gomphotherium*). Models were time constrained at 22 Ma to account for the physical connection between Africa and Eurasia after 22 Ma. A constraint considering the passage of Proboscidea to South America was also modelled for two ages: 3.5 Ma for a late passage (Figure S4) and 9 Ma for a possible early passage (Figure S5).

## Data Availability

• Raw sequencing reads have been uploaded to the SRA under BioProject SRA: PRJNA771914 and are publicly available as of the date of publication. The partial mitochondrial sequence of *Notiomastodon platensis* has been uploaded to GenBank under the accession numbers GenBank: OL631592 - OL631597 and is publicly available as of the date of publication. • This paper does not report original code. • Supplemental information (*Notiomastodon platensis* partial mtDNA sequence including missing data, BEAST xml for palaeogenetic analysis, BEAST xml file for total evidence analysis, morphological matrix, Total evidence tree file) has been deposited at Mendeley Data under https://doi.org/10.17632/msbxvxsdnh.1 and is publicly available as of the date of publication. Any additional information required to reanalyse the data reported in this paper is available from the lead contact upon request.
