# Supplementary material for: Revisiting proboscidean phylogeny and evolution through total evidence and palaeogenetic analyses including Notiomastodon ancient DNA
Source: iScience. 2021 Dec 4;25(1):103559. doi: 10.1016/j.isci.2021.103559 (PMC8693454; doi:10.1016/j.isci.2021.103559)
Supplement: Document S1. Figures S1–S6 and Tables S1–S3 [file mmc1.pdf]

iScience, Volume *25*

Supplemental information

Revisiting proboscidean phylogeny and evolution through total evidence and palaeogenetic analyses including *Notiomastodon* ancient DNA

Sina Baleka, Luciano Varela, P. Sebastián Tambusso, Johanna L.A. Paijmans, Dimila Mothé, Thomas W. Stafford Jr., Richard A. Fariña, and Michael Hofreiter

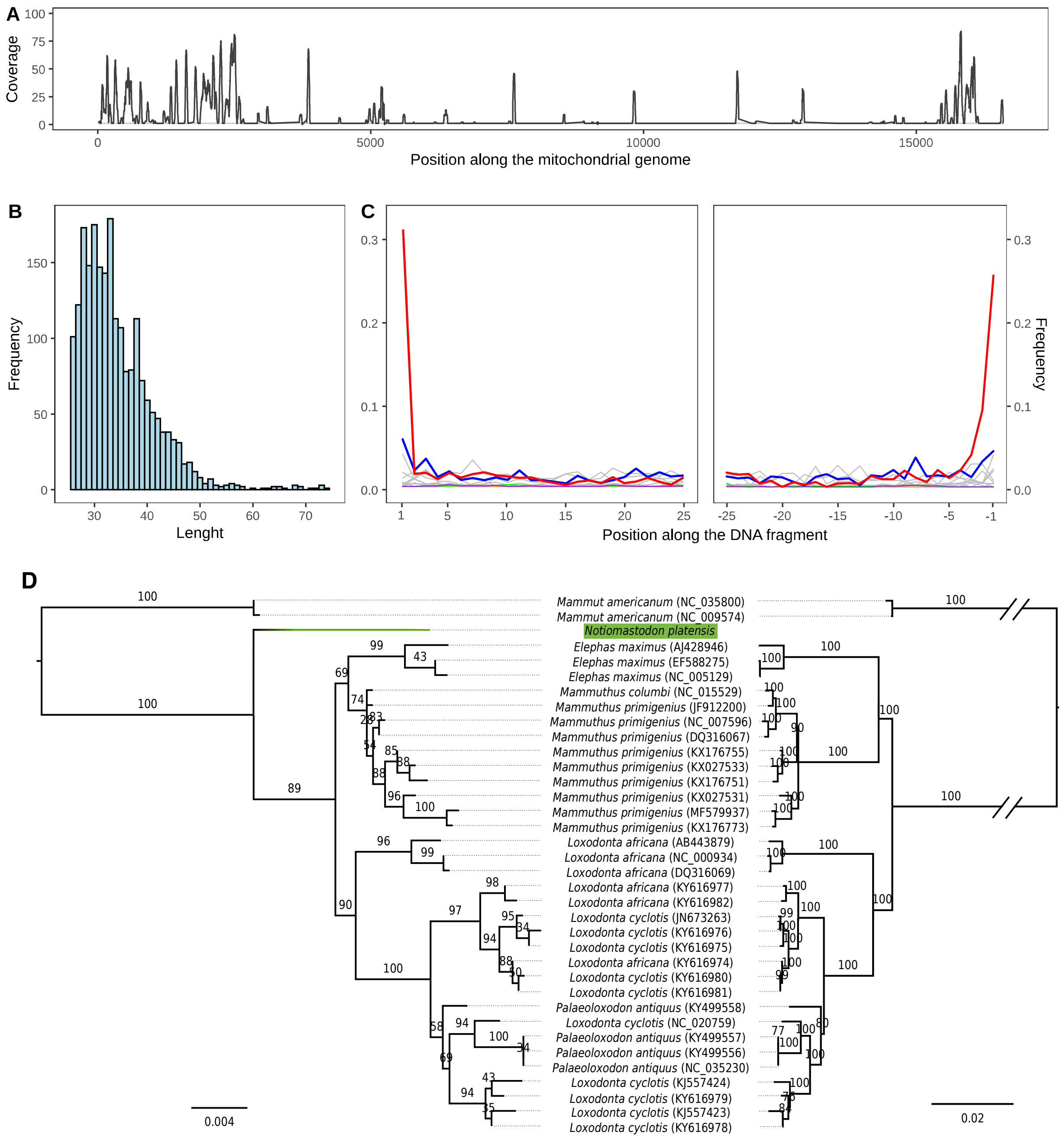

Figure S1: Overview of characteristics of the recovered *Notiomastodon* ancient DNA. Related to STAR Methods. A) Coverage of the mitochondrial genome, starting at the same position as NC_007596. B) Read length distribution of mapped reads. C) Frequency of C to T (red) and G to A (blue) misincorporations across mapped reads from the 5' (left) and 3' (right) end. D) Maximum-likelihood phylogenetic reconstruction of the position of *Notiomastodon platensis* compared to thirty-five extinct and extant proboscidean DNA sequences. The left part shows the phylogeny when including *Notiomastodon platensis* and removing columns with missing data (alignment length 2548 bp). The right part shows the phylogeny when the sequence of *Notiomastodon platensis* is excluded and thus the complete mitochondrial genome can be used (alignment length 16,026 bp). Node support is given as bootstrap values.

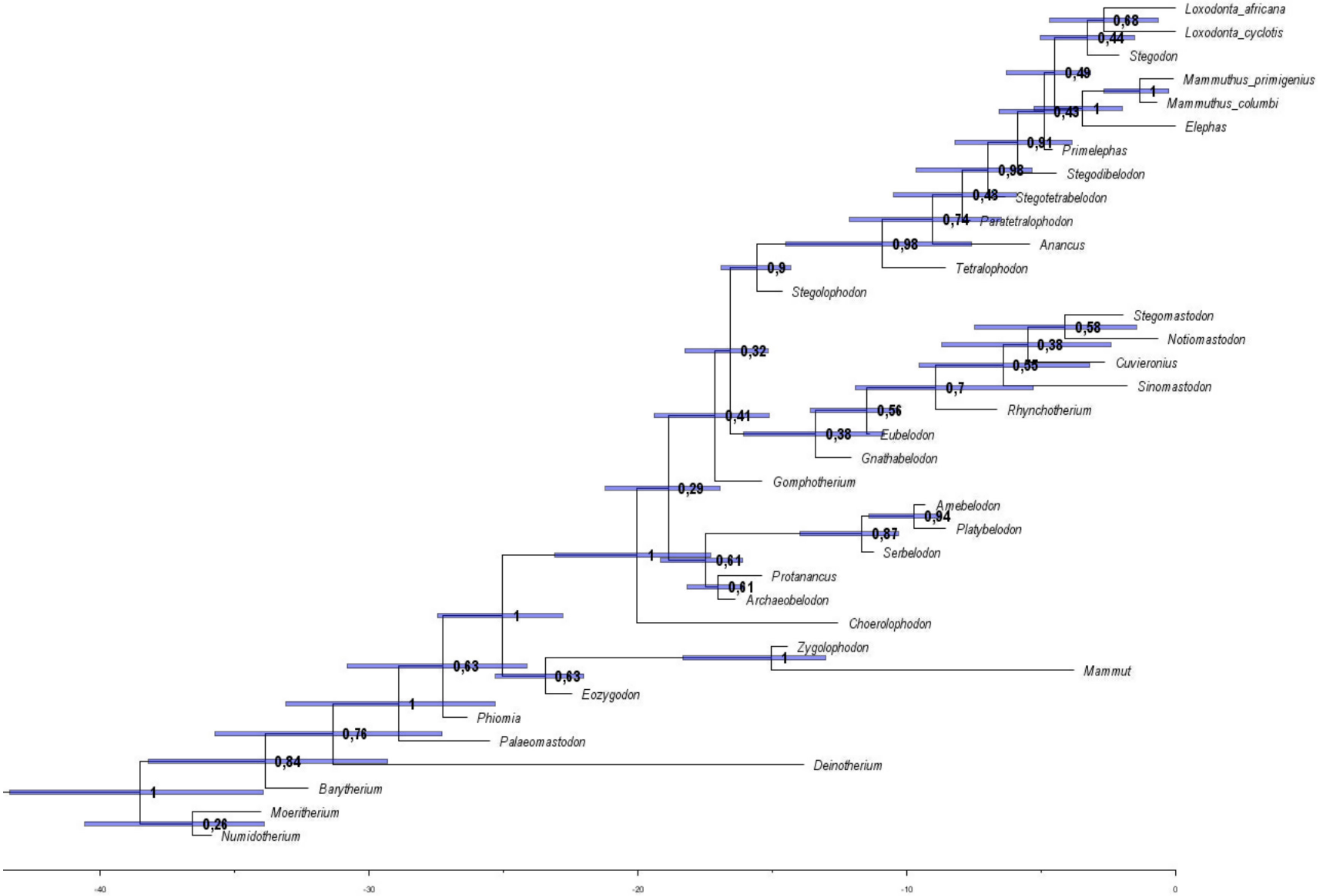

Figure S2: Total Evidence phylogenetic inference. Related to Figure 4. Complete output from the BEAST analysis including node support and divergence time estimates.

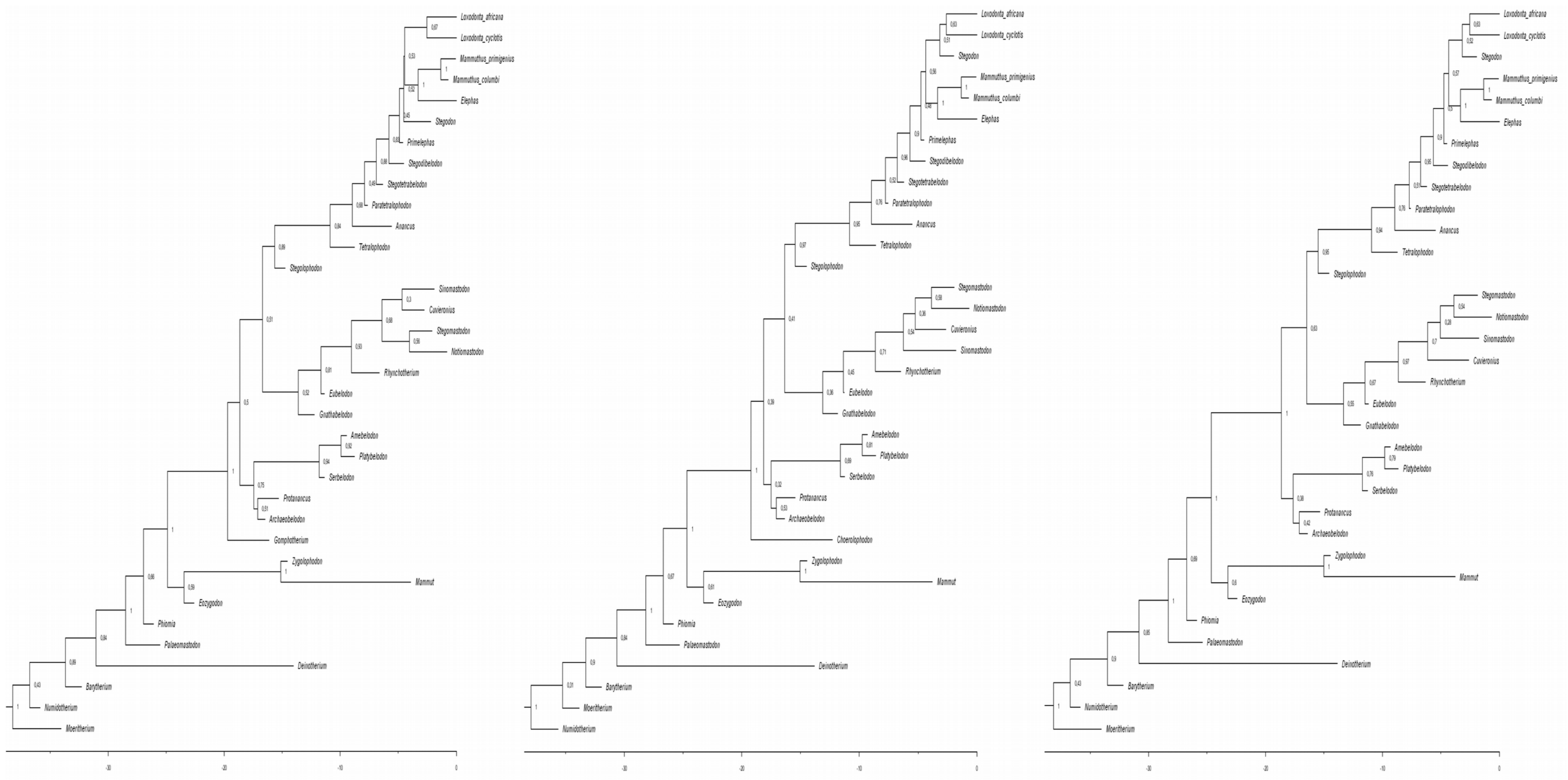

Figure S3: Total Evidence phylogenetic inference results after the exclusion of Choerolophodon and Gomphotherium. Related to Figure 4. Left: phylogeny after the exclusion of *Choerolophodon.* Centre: phylogeny after the exclusion of *Gomphotherium*. Right: phylogeny after the exclusion of both *Choerolophodon* and *Gomphotherium*.

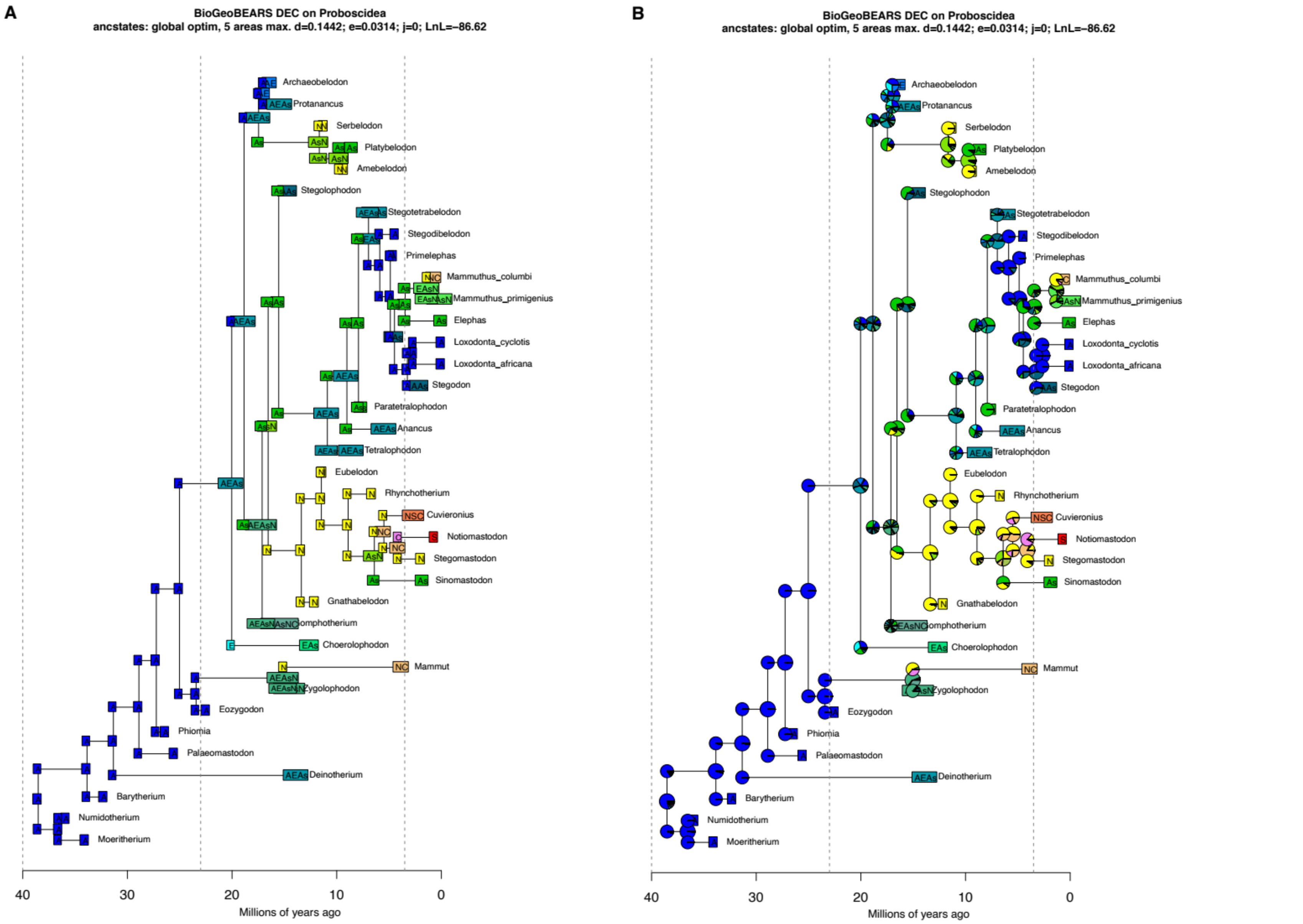

Figure S4: BioGeoBEARS output 3.5 Ma. Related to Figure 4. Complete output of the DEC model with time constraints at 22 Ma (connection between Africa and Eurasia) and 3.5 Ma (late passage of Proboscidea to South America), A) showing the single-most probable ancestral state/range, and B) pie

charts showing ancestral state/range probabilities on each node. It is worth noting that the single-most probable ancestral state/range at a particular node could still have very low probability.

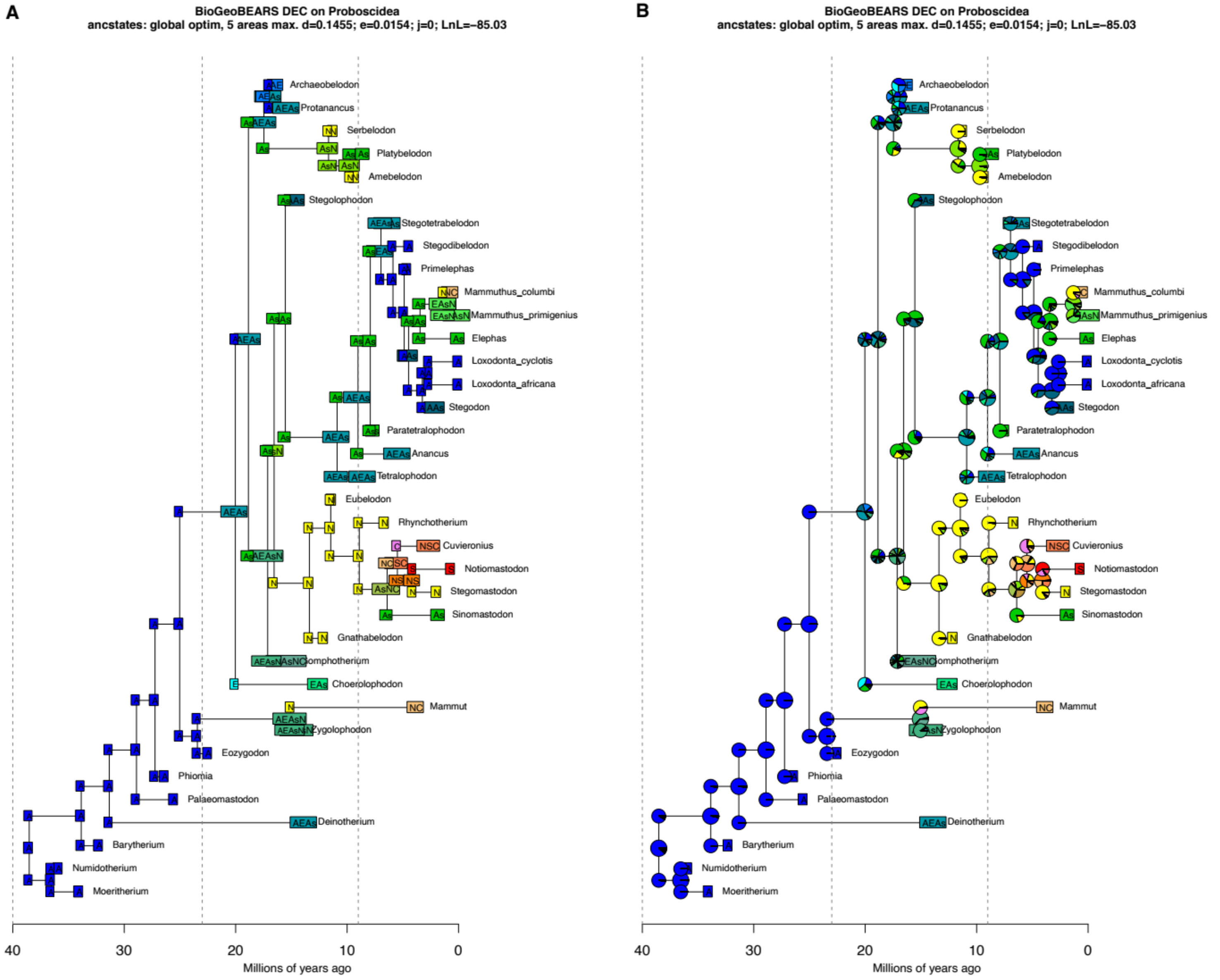

Figure S5: BioGeoBEARS output 9 Ma. Related to Figure 4. Complete output of the DEC model with time constraints at 22 Ma (connection between Africa and Eurasia) and 9 Ma (early passage of Proboscidea to South America), A) showing the single-most probable ancestral state/range, and B) pie

charts showing ancestral state/range probabilities on each node. It is worth noting that the single-most probable ancestral state/range at a particular node could still have very low probability.

A

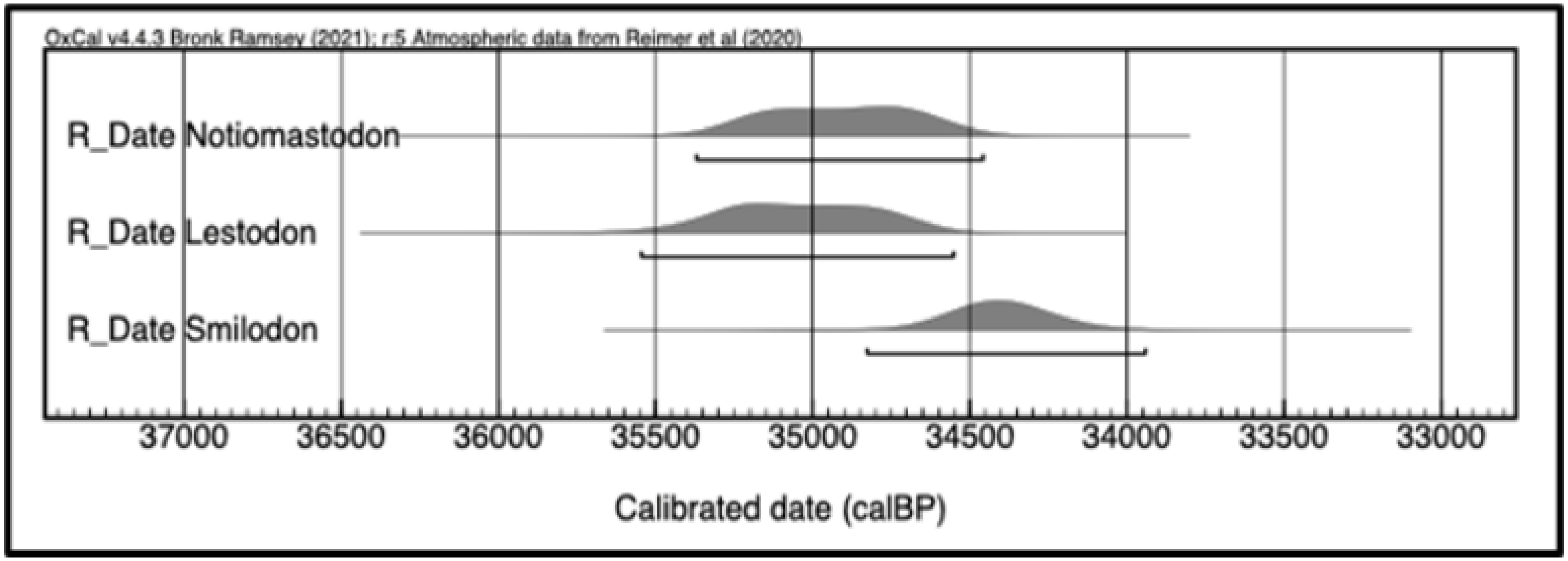

B

Sample No.	Museum No.	Taxon	Specimen	Description	AMS LAB No.	$^{14}C \pm 1$ SD RC yr. BP	CAL BP Range ± 2 SD (95.4% C.I.)	C/N (Atomic)	$\delta^{13}C$ ‰ (VPDB)	$\delta^{15}N$ ‰ (AIR)
SR-8820	CAV 499	*Notiomastodon platensis*	Tooth	RM[1]	UCIAMS-142845	30,510 ± 240	35,370-34,450	3.26	-18.7	+12.6
SR-8819	CAV 999	*Smilodon cf. populator*	Bone	R. Pre-maxilla	UCIAMS-142844	29,920 ± 230	34,830-33,940	3.29	-17.8	+13.6
SR-8823	CAV 645A	*Lestodon armatus*	Bone	Ulna	UCIAMS-142846	30,700 ± 240	35,550-34,550	3.29	-18.9	+10.8

Figure S6: Calibrated ages for *Notiomastodon*, *Lestodon*, and *Smilodon*. Related to STAR Methods. AMS ^{14}C measurements on ultrafiltration-purified collagen from *Notiomastodon platensis* and stratigraphically associated sabre tooth cat and giant ground sloth from Arroyo del Vizcaíno. ^{14}C measurements were made on the > 30 kDa ultrafiltration gelatin fraction. The proposed time range for accumulation of the site's animal remains is 35,550 to 33,940 cal BP, calibrated using OxCal 4.4.3 (17/2/2021) IntCal 20. A) Calibrated age distributions for the three tested samples. B) Values for each of the three samples tested.

	reads processed	too short reads	reads remaining	uncombined (if paired -end sequencing)	combined pairs (if paired-end sequencing)	Mapping to reference	Mapping after de-duplication	% after de-duplication
**Shotgun & Shotgun Blanks**								
STE1_lox	10,387,986	1,502,981	8,885,005	1,000,802	7,884,203	3	3	0.0000%
STE_UV7_lox	813,986	311,801	502,185			0	0	0.0000%
STE_UV1_lox	5,525,064	1,336,532	4,188,532	167,756	4,020,776	12	9	0.0002%
STE_CO1_lox	16,217,894	3,064,171	13,153,723	645,816	12,507,907	2,067	21	0.0002%
STE_B2_lox	43,498,444	14,376,564	29,121,880	1,605,958	27,515,922	1,082	8	0.0000%
Extraction Blank_lox	1,611,860	856,769	755,091			821	1	0.0001%
Library Blank_lox	8,063,559	5,054,356	3,009,203	14,648	2,994,555	0	0	0.0000%
**Capture (used for consensus sequence)**								
STE_cap2_lox	73,473,495	5,666,701	67,806,794	3,390,568	64,416,226	717	608	0.0009%
STE_cap2_mam						772	660	0.0010%
STE_cap2_mast						729	623	0.0010%
STE_CO2_cap2_lox	11,927,326	1,970,048	9,957,278	399,500	9,557,778	49,947	188	0.0020%
STE_CO2_cap2_mam						52,620	191	0.0020%
STE_CO2_cap2_mast						51,485	179	0.0019%
STE_UV2_cap2_lox	10,753,223	1,890,199	8,863,024	292,674	8,570,350	26,660	144	0.0017%
STE_UV2_cap2_mam						26,259	145	0.0017%
STE_UV2_cap2_mast						20,256	125	0.0015%
STE_UV3_cap2_lox	14,512,258	2,301,910	12,210,348	459,850	11,750,498	41,274	147	0.0013%
STE_UV3_cap2_mam						35,554	127	0.0011%
STE_UV3_cap2_mast						38,706	127	0.0011%
STE_UV4_cap2_lox	13,346,370	2,019,838	11,326,532	427,807	10,898,725	11,107	99	0.0009%
STE_UV4_cap2_mam						11,850	104	0.0010%
STE_UV4_cap2_mast						10,790	91	0.0008%

STE_CO3+4_cap2_lox	8,719,101	751,416	7,967,685			12,896	325	0.0041%
STE_CO3+4_cap2_mam						13,291	350	0.0044%
STE_CO3+4_cap2_mast						12,698	334	0.0042%
STE_UV5+6_cap2_lox	9,463,672	786,342	8,677,330			1,394	111	0.0013%
STE_UV5+6_cap2_mam						1,356	114	0.0013%
STE_UV5+6_cap2_mast						1,451	114	0.0013%
STED1_cap2_lox	4,645,008	263,060	4,381,948			1,645	86	0.0020%
STED1_cap2_mam						1,788	89	0.0020%
STED1_cap2_mast						1,720	80	0.0018%
STED3_cap2_lox	6,300,248	475,184	5,825,064			1,542	250	0.0043%
STED3_cap2_mam						1,643	270	0.0046%
STED3_cap2_mast						1,570	255	0.0044%
STED4_cap2_lox	5,366,544	262,257	5,104,287			1,075	177	0.0035%
STED4_cap2_mam						1,166	190	0.0037%
STED4_cap2_mast						1,279	195	0.0038%
**Capture (not used for consensus) & captured blanks**								
STE_B1_cap2_lox	10,949,180	2,020,470	8,928,710	332,015	8,596,695	44,122	13	0.0002%
STED2_cap2_lox	5,526,397	1,103,470	4,422,927			2,302	15	0.0003%
Extraction Blank_1_lox	1,483,183	700,741	782,442			3260	3	0.0004%
Library Blank_1_lox	1,724,971	625,303	1,099,668			1	1	0.0001%
Extraction Blank_2_lox	2,402,045	1,166,610	1,235,435			4,635	6	0.0005%
Library_Blank_2_lox	Even after reamplification no visible product							

Table S1: Sequencing statistics of the ten libraries that were combined to reconstruct the consensus sequence. Related to STAR Methods. Each library was mapped to *Loxodonta africana* (indicated by the addition of "_lox" to the library name in column one), *Mammuthus primigenius* ("_mam") and the American mastodon *Mammut americanum* ("_mast").

Divergence time *Notiomastodon*	fossil priors	prior distribution	Broad/Narrow
Mean (95% HPD)			
13.7 (10.1-17.5)	All 3	uniform	broad
14.1 (10.2-18.1)	All 3	uniform	narrow
12.91 (9.4-16.9)	All 3	lognormal	broad
13.3 (9.96-16.8)	All 3	lognormal	narrow
13.4 (9.7-17.2)	All 3	normal	broad
13.5 (10-17.2)	All 3	normal	narrow
13.5 (10.1-17)	2 (Ele-Mast, Lox-Eur)	lognormal	narrow
13.9 (10.1-17.7)	2 (Ele-Mast, Asian-Mam)	lognormal	narrow
11.8 (8.7-14.3)	2 (Asian-Mam, Lox-Eur)	lognormal	narrow
14.9 (10.4-19.4)	Elephantid-Mastodon	Uniform	narrow/broad
14.6 (10.4-18.89)	Elephantid-Mastodon	lognormal	narrow/broad
14.9 (10.5-19.6)	Elephantid-Mastodon	normal	narrow/broad
11.8 (8.1-15.8)	Loxodonta-Eurasian	Uniform	narrow
11.9 (8.6-15.7)	Loxodonta-Eurasian	lognormal	narrow
11.7 (7.8-16.1)	Loxodonta-Eurasian	normal	narrow
10.96 (6.4-16)	Asian-Mammoth	Uniform	narrow
11.5 (7.3-16.5)	Asian-Mammoth	lognormal	narrow
10.7 (5.7-16.2)	Asian-Mammoth	normal	narrow

Table S2: Divergence times between *Notiomastodon* and the Elephantidae based on ancient DNA. Related to STAR Methods. Mean divergence time as well as 95% highest posterior density of different combinations of one, two, or three fossil calibrations, as well as uniform, normal, or lognormal prior distribution, and the “narrow” (Brandt et al., 2012) or “broad” (Rohland et al., 2010) calibration of fossils. Elephantid-Mastodon: split between the American mastodon (genus *Mammut*) and all other genera used in this analysis (there is only one calibration, which was used in both the narrow and the broad analysis); Loxodonta-Eurasian: split between *Loxodonta/Palaeoloxodon* and the two Eurasian genera *Elephas* and *Mammuthus;* Asian-Mammoth: split between the Asian elephant (*Elephas*) and the mammoth (*Mammuthus*).

OTUs	LAD	FAD	Africa	Europe	Asia	North America	South America
Moeritherium	28.4	40.4	1	0	0	0	0
Numidotherium	33.9	55.8	1	0	0	0	0
Barytherium	28.4	37.2	1	0	0	0	0
Deinotherium	2	15.97	1	1	1	0	0
Palaeomastodon	23.03	28.4	1	0	0	0	0
Phiomia	23.03	40.4	1	0	0	0	0
Eozygodon	22	23	1	0	0	0	0
Zygolophodon	13	18	1	1	1	1	0
Mammut	0.011	5.3	0	0	0	1	0
Choerolophodon	7.246	19	0	1	1	0	0
Gomphotherium	0.012	23.03	1	1	1	1	0
Amebelodon	8.7	10.3	0	0	0	1	0
Platybelodon	5.332	20.4	0	0	1	0	0
Serbelodon	10.3	13.6	0	0	0	1	0
Protanancus	11.608	19	1	1	1	0	0
Archaeobelodon	15.97	16.9	1	1	0	0	0
Gnathabelodon	10.3	13.6	0	0	0	1	0
Sinomastodon	0.781	2.588	0	0	1	0	0
Eubelodon	10.3	13.6	0	0	0	1	0
Rhynchotherium	3.6	11.608	0	0	0	1	0
Stegomastodon	0.03	4.9	0	0	0	1	0
Notiomastodon	0.006	1.2	0	0	0	0	1
Cuvieronius	0.012	4.9	0	0	0	1	1
Tetralophodon	2.588	11.608	1	1	1	0	0
Anancus	0.126	7.246	1	1	1	0	0
Paratetralophodon	5.9	11.6	0	0	1	0	0
Stegolophodon	14	18	1	0	1	0	0
Stegodon	0.012	5.332	1	0	1	0	0
Stegotetrabelodon	5.332	11.1	1	1	1	0	0
Stegodibelodon	3.6	5.332	1	0	0	0	0
Primelephas	3.6	7.246	1	0	0	0	0
Loxodonta africana	-	-	1	0	0	0	0
Loxodonta cyclotis	-	-	1	0	0	0	0
Elephas	-	-	0	0	1	0	0
Mammuthus primigenius	0.004	0.15	0	1	1	1	0
Mammuthus columbi	0.011	1.5	0	0	0	1	0

Table S3: Stratigraphic ranges of fossil taxa & geographic ranges of fossil and extant taxa. Related to Figure 4. First and last occurrence records in million years for the fossil taxa used in the total evidence analysis and geographic ranges for all proboscidean taxa used in the historical biogeography analysis.
